# Random peptide mixtures as new crop protection agents

**DOI:** 10.1111/1751-7915.13258

**Published:** 2018-02-28

**Authors:** Shiri Topman, Dafna Tamir‐Ariel, Heli Bochnic‐Tamir, Tal Stern Bauer, Sharoni Shafir, Saul Burdman, Zvi Hayouka

**Affiliations:** ^1^ Institute of Biochemistry, Food Science and Nutrition The Robert H. Smith Faculty of Agriculture, Food and Environment The Hebrew University of Jerusalem Rehovot 76100 Israel; ^2^ Department of Plant Pathology and Microbiology The Robert H. Smith Faculty of Agriculture, Food and Environment The Hebrew University of Jerusalem Rehovot 76100 Israel; ^3^ Department of Entomology The Robert H. Smith Faculty of Agriculture, Food and Environment The Hebrew University of Jerusalem Rehovot 76100 Israel

## Abstract

Many types of crops are severely affected by at least one important bacterial disease. Chemical control of bacterial plant diseases in the field vastly relies on copper‐based bactericides, yet with limited efficacy. In this study, we explored the potential of two random peptide mixture (RPM) models as novel crop protection agents. These unique peptide mixtures consist of random combination of l‐phenylalanine and l‐ or d‐lysine (FK‐20 and FdK‐20, respectively) along the 20 mer chain length of the peptides. Both RPMs displayed powerful bacteriostatic and bactericidal activities towards strains belonging to several plant pathogenic bacterial genera, for example, *Xanthomonas*,* Clavibacter* and *Pseudomonas. In planta* studies in the glasshouse revealed that RPMs significantly reduced disease severity of tomato and kohlrabi plants infected with *Xanthomonas perforans* and *Xanthomonas campestris* pv. *campestris* respectively. Moreover, RPM effects on reduction in disease severity were similar to those exerted by the commercial copper‐based bactericide Kocide 2000 that was applied at a 12‐fold higher concentration of the active compound relative to the RPM treatments. Importantly, the two tested RPM compounds had no toxic effect on survival of bees and Caco‐2 mammalian cells. This study demonstrates the potential of these innovative RPMs to serve as crop protection agents against crop diseases caused by phytopathogenic bacteria.

## Introduction

Agricultural yields are highly damaged by plant diseases caused by microorganisms. Plant pathogenic bacteria are among the most important causal agents of plant diseases with almost all major crops being severely affected by one or more important bacterial diseases (Mahlein *et al*., 2012). The chemical control options to cope with bacterial diseases in agriculture are limited, and the strategies available for their management are often inadequate (Gitaitis and Walcott, 2007). To date, chemical control of bacterial plant diseases in the field predominantly relies on copper (Cu)‐based bactericides (Datta *et al*., 2015). These products, however, possess limited efficacy. Copper‐based bactericides possess relatively low solubility and are easily washed from the plant surface by rain or overhead irrigation, conditions that are highly conducive to many bacterial plant diseases (De La Fuente and Burdman, 2011). Under these conditions, frequent sprays of copper‐based bactericides are required, increasing production costs as well as environmental contamination, without necessarily providing adequate protection. Moreover, frequent sprays with relatively high concentrations of Cu might cause phytotoxicity and often lead to emergence of Cu‐resistant strains (Hancock, 2001; Behlau *et al*., 2011; Worthington *et al*., 2012). Therefore, there is an urgent need to develop novel technologies to manage and prevent bacterial plant diseases and prevent food loss.

In recent years, much attention has been paid to the search of new classes of antibiotics to tackle the bacterial resistance crisis. Host defence peptides (HDPs) have emerged as potential candidates (Zasloff, 2002; Diamond *et al*., 2009; Rathinakumar *et al*., 2009). The modes of action of HDPs vary among different types; however, many HDPs have been shown to possess the ability to disrupt bacterial membrane (Oren and Shai, 1997; Oren *et al*., 2002; Zasloff, 2002; Rathinakumar *et al*., 2009). Some HDPs display a characteristic selectivity, favouring attack on prokaryotic membranes relative to eukaryotic ones (Hancock, 2001; Rathinakumar *et al*., 2009). Moreover, cationic HDPs are able to attack a variety of pathogens, including most Gram‐negative and Gram‐positive bacteria, fungi, enveloped viruses and eukaryotic parasites (Mukherjee and Hooper, 2015).

Natural HDPs have also been studied as potential crop protection agents. HDPs such as magainin II, cecropin B and MSI‐99 have been shown to have a strong inhibitory effect *in vitro* against some important plant pathogenic bacteria such as strains belonging to the *Pseudomonas, Clavibacter and Xanthomonas* genera and fungi including *Alternaria solani* and *Penicillium digitatum* (Alan and Earle, 2002). However, several studies revealed that some HDPs might have undesired characteristics such as low stability, non‐specific toxicity and poor bioavailability (Marcos *et al*., 2008), all of these limiting their potential applicability for crop protection.

Several reports have shown that different sequence‐specific, synthetic HDP derivatives display antimicrobial activity towards plant pathogenic bacteria. For instance, synthetic linear undecapeptide derivatives based on the peptide KKLFKKILKFL‐NH2 (BP76) were able to inhibit growth of various plant pathogenic bacteria including *Erwinia amylovora* and *Pseudomonas syringae in vivo* and to reduce *E. amylovora* infection in detached apple and pear flowers (Badosa *et al*., 2007). It is also possible to replace l‐amino acids by d‐amino acids to enhance peptide stability against protease degradation and/or to reduce its haemolytic activity without affecting the antimicrobial effect (Güell *et al*., 2011). Although these findings are promising, these synthetic peptides present two major disadvantages: (i) the ability of bacteria to develop resistance towards them (Dobson *et al*., 2014) and (ii) the high cost of their production and purification.

The broad molecular diversity among HDPs suggests that their prokaryotic‐selective activity is not tightly associated with specific features of the amino acid sequence or peptide conformation (Rathinakumar *et al*., 2009). Based on this knowledge, Hayouka *et al*. (2013) recently introduced the concept of random peptide mixtures (RPMs) using a modification of the conventional Fmoc‐based solid‐phase synthesis method: instead of using one pure amino acid at each coupling step, a mixture of two amino acids in a defined proportion is used through the synthesis process. The result is a 2^n^ (where n is the peptide chain length) sequences of random peptides with a defined composition and controlled chain length. This novel synthetic type of peptides could overcome the disadvantages that characterize sequence‐specific, synthetic HDPs.

Based on the aforementioned notion, a variety of RPMs comprised of hydrophobic and cationic α‐amino acids was developed and showed strong antimicrobial activity towards both Gram‐negative and Gram‐positive bacteria, including antibiotic‐resistant bacteria (Hayouka *et al*., 2013). A subsequent study showed that FK‐20 (a 20 mer random peptide mixture comprised of random sequences of l‐phenylalanine and l‐lysine) and FdK‐20 (a 20 mer peptide comprised of random peptide sequences of l‐phenylalanine and d‐lysine) have the abilities to inhibit the growth of planktonic methicillin‐resistant *Staphylococcus aureus* (MRSA), to inhibit MRSA biofilm formation and to eradicate mature MRSA biofilm (Stern *et al*., 2016). A further study suggested that these two RPMs possess different modes of action. While FK‐20 assembles into antimicrobial heterogeneous pore‐like structures on the membrane, FdK‐20 attacks bacterial membranes without oligomerizing into visible pores (Hayouka *et al*., 2017).

The objective of the current study was to explore the potential of RPMs as crop protection agents. We screened the ability of FK‐20 and FdK‐20 to inhibit growth as well as their bactericidal activity towards several important plant pathogenic bacteria. Then, we characterized the ability of these compounds to reduce disease severity *in planta*, using two pathosystems involving *Xanthomonas* species pathogenic to tomato and brassica plants. Further, we assessed whether the RPMs affect honeybees to evaluate possible effects of these compounds on insects in case of future field application, as well as on survival of Caco‐2 mammalian cells to assess possible toxic effects on humans. Overall, our findings suggest that FK‐20 and FdK‐20 RPMs have the potential to serve as novel crop protection agents.

## Results and discussion

### Bacteriostatic and bactericidal effects of RPMs

**Figure 1:**
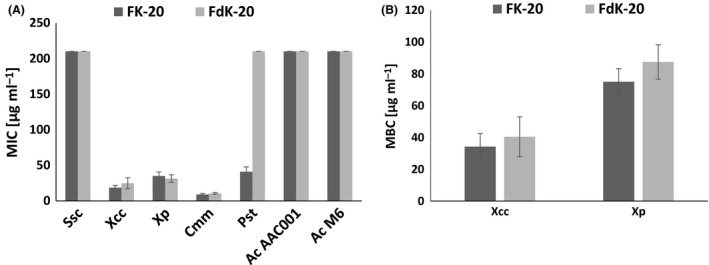
Bacteriostatic and bactericidal effects of RPMs towards several plant pathogenic bacteria. A. Growth inhibition activity (minimal inhibitory concentration, MIC) of FK‐20 and FdK‐20 RPMs towards different bacteria after 24 h incubation at 28°C: *Ssc*,* Streptomyces scabies* Av; *Xcc*,* Xanthomonas campestris* pv. *campestris* ATCC 33913; *Xp*,* Xanthomonas perforans* 97‐2; *Cmm*,* Clavibacter michiganensis* subsp. *michiganensis* NCPPB 382; *Pst*,* Pseudomonas syringae* pv. *tomato* DC3000; *Ac* AAC001, *Acidovorax citrulli* AAC00‐1; and *Ac* M6, *A. citrulli* M6. B. Bactericidal effect (minimal bactericidal concentration, MBC) of FK‐20 and FdK‐20 RPMs towards *Xcc* ATCC 33913 and *X. perforans* 97‐2 after 24 h incubation at 28°C. The highest concentration tested was 200 μg ml^−1^; therefore, values ~200 μg ml^−1^ mean that no inhibitory effect was detected under tested conditions. Data represent averages and standard errors of at least three independent experiments with three replicates for each peptide/bacterial strain combination.

We assessed the ability of homochiral and heterochiral random mixtures of phenylalanine (F) and lysine (K) (FK‐20 and FdK‐20, respectively), to inhibit the growth of several plant pathogenic bacteria. The minimal inhibitory concentration (MIC) of these RPMs was tested towards several strains belonging to the genera *Streptomyces* and *Clavibacter* (Gram‐positive) and *Xanthomonas*,* Pseudomonas* and *Acidovorax* (Gram‐negative; Table 1). Both RPMs were found to possess strong bacteriostatic activity towards *Xanthomonas campestris* pv. *campestris* (*Xcc*), *Xanthomonas perforans (Xp)* and *Clavibacter michiganensis* subsp. *michiganensis* (*Cmm*), with MIC values ranging from ~9 to 35 μg ml^−1^ (Fig. 1A). In contrast, both compounds were not effectively active against *Streptomyces scabies* and the two tested strains of *Acidovorax citrulli*, representing the two major groups of this pathogen (M6, representing group I strains, isolated mainly from non‐watermelon cucurbits, and AAC00‐1, representing group II strains, isolated mainly from watermelon). These results indicate that the antibacterial activity of RPMs does not depend on Gram‐stain group belonging.

**Table 1:** Bacterial strains used in this study

Pathogen	Strain	Gram	Disease	Abbreviation	Source
*Acidovorax citrulli*	M6, AAC00‐1	(−)	Bacterial fruit blotch of cucurbits	*Ac* M6, *Ac* AAC00‐1	Walcott *et al*. (2000) and Burdman *et al*. (2005)
*Xanthomonas campestris* pv. *campestris*	ATCC 33913	(−)	Black rot disease of crucifer plants	*Xcc*	da Silva *et al*. (2002)
*Xanthomonas perforans*	97‐2	(−)	Bacterial spot disease of tomato and pepper	*Xp*	Jones *et al*. (2004)
*Pseudomonas syringae* pv. *tomato*	DC3000	(−)	Bacterial spot disease of tomato	*Pst*	Cuppels (1986)
*Clavibacter michiganensis* subsp. *michiganensis*	NCPPB 382	(+)	Bacterial canker and wilt of tomato	*Cmm*	Meletzus and Eichenlaub (1991)
*Streptomyces scabies*	Av	(+)	Potato common scab	*Ssc*	Burdman lab collection

The observed differences in antimicrobial activity of the RPMs towards the tested bacteria could be attributed to differences in membrane components such as lipid composition or membrane charge that have crucial effects on the interaction between antimicrobial peptides (AMPs) and the bacterial membrane (Güell *et al*., 2011). Interestingly, while FK‐20 had a significant bacteriostatic activity towards *Pseudomonas syringae* pv. *tomato* (*Pst*; MIC value of ~40 μg ml^−1^), the MIC value for the heterochiral FdK‐20 was above 200 μg ml^−1^ (Fig. 1A). These differences could be due to the different modes of action of the homo‐ versus heterochiral RPMs as described recently (Ryadnov *et al*., 2017).

Minimal bactericidal concentration (MBC) assays were carried out on the two tested strains of *Xanthomonas* (*Xcc* and *X. perforans*). These assays revealed that both FK‐20 and FdK‐20 possess high bactericidal activity on the two strains, with MBC values ranging between ~30 and 90 μg ml^−1^ (Fig. 1B). Cationic peptides with specific sequences usually have MIC values that are equal to or differ by up to twofold from the MBC values (Hancock, 2001). Accordingly, our results show that there is an average of ~twofold differences between MIC and MBC values observed for *Xcc* and *X. perforans* in response to FK‐20 and FdK‐20. Interestingly, the *Xcc* strain appeared to be more sensitive to both FK‐20 and FdK‐20 than the *X. perforans* strain, showing MBC values that were about half of those observed for the latter. These findings support the results from MIC experiments that also revealed slightly lower MIC values for the *Xcc* relative to the *X. perforans* strain (Fig. 1A).

### Assessment of RPM antimicrobial activity in planta

To assess the potential of FK‐20 and FdK‐20 RPMs as crop‐protecting agents, we carried out *in planta* experiments using the *Xcc*–kohlrabi and the *X. perforans*–tomato pathosystems. In addition of the promising results in *in vitro* assays observed for *Xcc* and *X. perforans*, these are pathogens with significant impact on agriculture and well‐investigated models of plant pathogenic bacteria. *Xcc* is the causal agent of black rot of crucifers, one of the most threatening diseases of *Brassica* crops worldwide (Vicente and Holub, 2013). *X. perforans* is one among four *Xanthomonas* species (formerly collectively referred to as *X. campestris* pv. *vesicatoria*) causing bacterial spot disease, one of the major diseases of tomatoes in many parts of the world (Potnis *et al*., 2015). While Cu‐based bactericides are widely used to control black rot and bacterial spot diseases, the efficiency of this strategy is limited and emergence of Cu‐resistant *Xcc* and *X. perforans* strains occurs in the field under continuous application of Cu compounds (Jones *et al*., 1991; Lugo *et al*., 2013).

**Figure 2:**
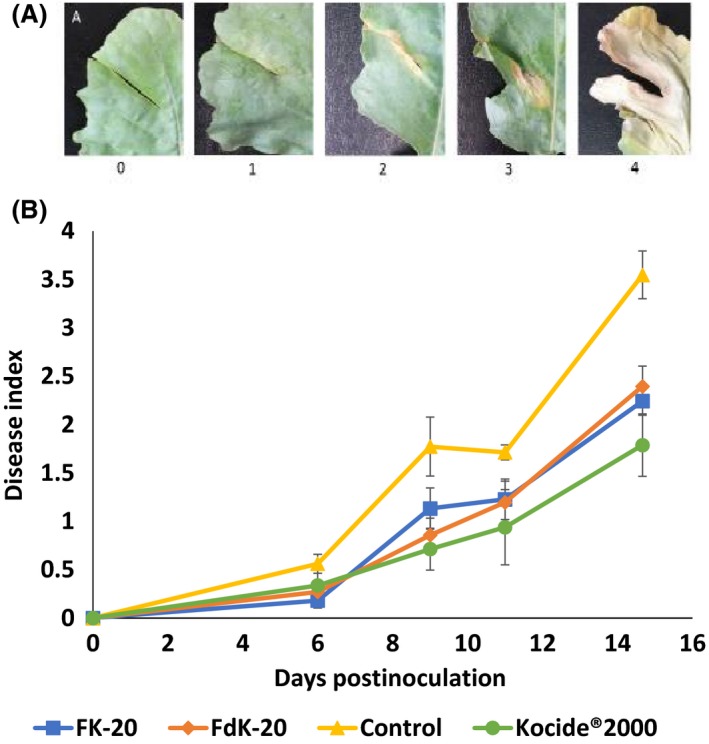
Effect of RPMs on black rot disease of kohlrabi caused by *Xanthomonas campestris* pv. *campestris* (*Xcc*). A. Black rot disease severity scale (also described in Experimental procedures). B. Disease progress over time. The three youngest leaves of ~4‐week‐old kohlrabi cv. Konmar plants were cut in two peripheral zones with scissors that were previously dipped in RPM solutions (FK‐20 or FdK‐20; 200 μg ml^−1^), Kocide^®^ 2000 (2500 μg ml^−1^) or sterilized double‐distilled water (DDW) followed by application of *Xcc* ATCC 33913 at 10^6^ CFU ml^−1^. Results represent averages and standard errors from three independent experiments, with four replicates per treatment in each experiment.

The whole plant is a much complex system than *in vitro* assays, and the need to increase the active compound concentration *in planta* relative to *in vitro* conditions has been reported (Güell *et al*., 2011). Therefore, in *in planta* experiments, we used a higher concentration of RPMs (200 μg ml^−1^) relative to the MIC values determined for these bacteria. No visible phytotoxic effects were observed in leaves of tomato and kohlrabi plants treated with such concentrations of FK‐20 and FdK‐20 in all experiments, suggesting that, under tested conditions, these RPMs do not affect plant health (data not shown). Pretreatment of kohlrabi leaves with FK‐20 and FdK‐20 significantly (*P* < 0.0001) reduced disease severity caused by inoculation with *Xcc* relative to control plants that were pretreated with sterilized double‐distilled water (DDW). The reduction in disease severity by both RPMs was comparable with that exerted by pretreatment with the copper hydroxide‐based commercial bactericide Kocide 2000 (DuPont; Fig. 2B).

**Figure 3:**
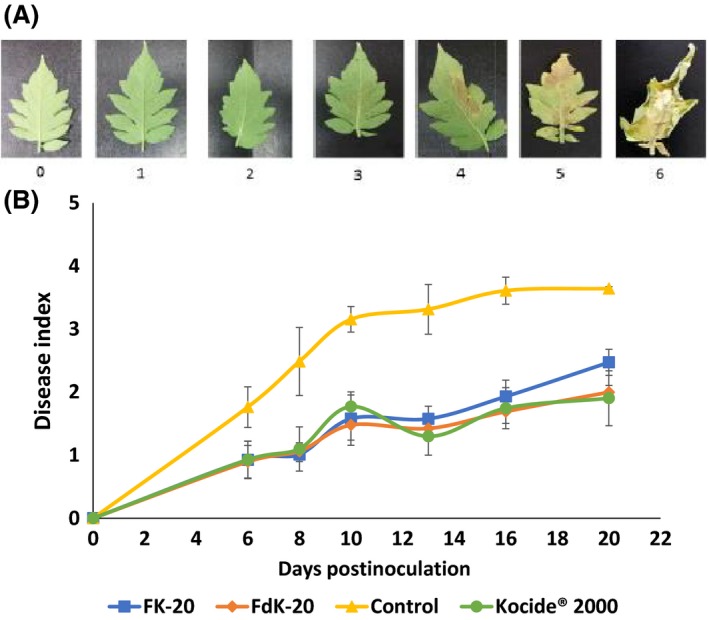
Effect of RPMs on bacterial spot disease of tomato caused by *Xanthomonas perforans*. A. Bacterial spot disease of tomato severity scale (also described in Experimental procedures). B. Disease progress over time. The five external leaflets of the three youngest, fully expanded leaves of ~6‐week‐old tomato cv. FA‐144 plants were pretreated by spraying both sides of the leaflets with RPMs (FK‐20 or FdK‐20; 200 μg ml^−1^), Kocide^®^ 2000 (2500 μg ml^−1^) or sterilized DDW. After 24 h, each leaflet was sprayed with *X*. *perforans* 97‐2 at 10^8^ CFU ml^−1^. Results represent averages and standard errors from three independent trials, with six replicates per treatment in each experiment.

As similar as observed for the kohlrabi–*Xcc* system, pretreatment of tomato leaves with the same RPMs at 200 μg ml^−1^ significantly (*P* < 0.0001) reduced bacterial spot symptoms caused by *X. perforans* relative to non‐pretreated controls (Fig. 3B). For instance, at 20 days postinoculation (dpi), leaves of tomato plants pretreated with FK‐20 and FdK‐20 showed average disease severity index of 2.4 and 1.9, respectively, compared to an average of 3.7 observed for control plants. Also, similarly to the kohlrabi–*Xcc* system, no significant differences were observed between RPMs and Kocide treatment (Fig. 3B).

Despite the different modes of infection and colonization of *Xcc* and *X. perforans* [penetration through hydathodes or wounds and vascular colonization in the case of *Xcc* (Vicente and Holub, 2013), and entering via stomata or wounds and colonization of the extracellular apoplast in the case of *X. perforans* (Potnis *et al*., 2015), chemical control of both pathogens mostly relies on repeated application of Cu‐based bactericides at high concentrations. Our experiments revealed that both FK‐20 and FdK‐20 had similar effects than commercial Kocide in terms of disease reduction, but at substantially lower (~12‐fold) concentrations of the active compounds.

Model membrane studies suggest that an essential step for the antibacterial activity of cationic peptides is their interaction with the bacterial cytoplasmic membrane and the subsequent distortion of the outer membrane in the case of Gram‐negative bacteria (Hancock, 2001). Furthermore, it was suggested that cationic peptides can attack secondary targets, which might explain, at least partially, why development of resistance against cationic peptides is relatively challenging (Hancock, 2001). As the FK‐20 and FdK‐20 RPMs contain ~millions (2^20^) of different peptide sequences, we assume that development of resistance towards RPMs would be even more difficult; however, this hypothesis should be verified in the future.

### Effects of RPMs on honeybees

Almost 90% of commercial pollination in agriculture is performed by honeybees (*Apis mellifera*), making them the most important commercial pollinators worldwide. The bee's population is decreasing dramatically in Europe and North America, with one of the reasons being the extensive use of pesticides (Genersch, 2010; Henry *et al*., 2012). Therefore, it is important to evaluate the effects of every chemical control treatment, including RPMs, on honeybees. Such evaluation is also important for broad estimation of general effects of RPM compounds on the environment. A no‐choice dietary feeding study was conducted using two relatively high concentrations (100 and 200 μg ml^−1^) of FK‐20 and FdK‐20. The survival of the bees was estimated, and the effect on sugar solution and pollen consumption was evaluated.

**Figure 4:**
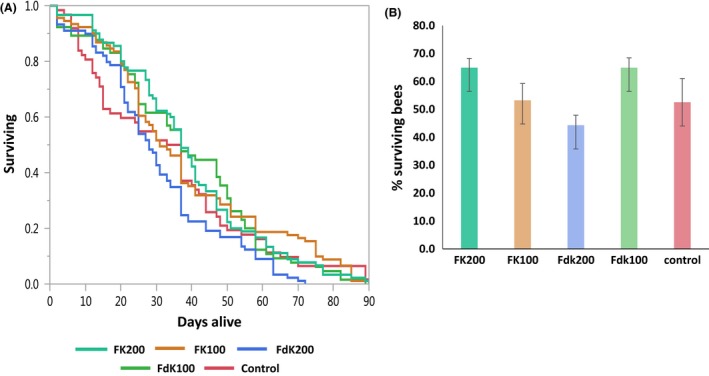
Assessment of RPMs effects on honeybees in a no‐choice dietary feeding study. Newly emerged bees were placed in Perspex cages with access to pollen and sugar solutions containing FK‐20 or FdK‐20 at 100 or 200 μg ml^−1^, or without RPMs (control). Three replicate cages were assigned to each diet treatment, with 60 to 90 randomly selected bees per treatment from six source colonies. A. Survival plot over time. No significant differences in bee's survival were detected among the treatments. B. Percentage (average and standard errors) of bees alive in day 30 of the experiment. No significant differences were detected among treatments.

At day 30 of the experiment, no significant differences were observed in the percentage of live bees between the two RPM treatments (*P* = 0.621). Importantly, survival analysis showed no significant differences between RPM treatments and the control group, supporting that FK‐20 and FdK‐20 have no toxic effects on bees, even at the relatively high concentrations (Fig. 4) used in *in planta* experiments (200 μg ml^−1^). Furthermore, there was no significant difference in sugar solution consumption or pollen consumption (data not shown), suggesting that RPMs had no antagonizing effects on taste‐wise of the bees and did not affect their need for protein source.

### Cytotoxicity towards mammalian cells

**Figure 5:**
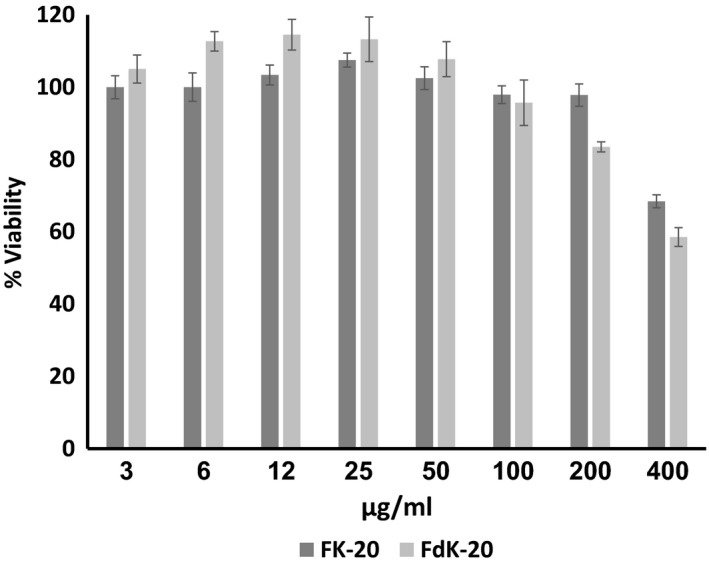
Caco‐2 MTT assay. The cytotoxic potential of FK‐20 and FdK‐20 was determined following incubation of exponentially growing Caco‐2 cells using the MTT assay. After 24 h of incubation, cells were washed and the percentage of viable cells (average and standard error) was determined in a plate reader. Percentage of cell viability values were determined as the average obtained from three independent experiments, with two replicates per RPM type and concentration in each experiment.

As a crop protection agent, it is important to determine the influence of the RPMs on the environment as well as on animal/human health. In the present study, we assessed the cytotoxicity potential of the FK‐20 and FdK‐20 RPMs towards mammalian cells by carrying out MTT (3‐(4,5‐dimethylthiazol‐2‐yl)‐2,5‐diphenyltetrazolium bromide) assays on Caco‐2 (heterogeneous human epithelial colorectal adenocarcinoma) cells. Results of these experiments are summarized in Figure 5. Caco‐2 cells showed no toxicity in response to concentrations of up to 100 μg ml^−1^ of FdK‐20 and up to 200 μg ml^−1^ of FK‐20. In these experiments, viability values slightly higher than 100% could be result of defence responses of the cells and/or due to non‐uniform dispersion of the cells in the wells. These findings suggest that the tested Fk‐20 could be safely used at up to 200 μg ml^−1^, while FdK‐20 could be used at up to 100 μg ml^−1^ for crop protection purposes, although it is plausible to assume that higher concentration than those will still be safe considering that *in vitro* and cell culture assays are more sensitive than exposure of whole organisms to these compounds at residual concentrations.

### Feasibility of RPMs as crop protection agents

One of the main advantages of the RPM approach is the simplicity and relatively low cost of RPM synthesis, mainly because there is no need of HPLC purification. In this regard, while the production of one gram of natural antimicrobial peptides with specific sequences cost hundreds of dollars, the cost of the synthesis of one gram of RPMs like those used in this study is just a few dollars (~US$ 1–2). In terms of economic feasibility, this makes RPMs a competitive technology. For instance, the estimated cost for one single treatment of Kocide to manage bacterial spot disease is about US$ 15 per acre (calculation based on product price in the market and a recommended dosage of 2.5 g Kocide per ml). A similar treatment but with RPMs, at a dosage of 200 μg ml^−1^ (much lower than that of Kocide), would demand ~4 g of RPMs, whose production cost would be around US$ 4–8. While these numbers only reflect the expected cost of synthesis of the active compound, this comparison highlights the economic competitiveness of the RPM technology. With that said, we are currently developing a variety of chemical approaches to reduce even more the production costs of RPMs, while maintaining their antimicrobial activity.

An additional important issue regarding the feasibility of the approach is the stability of the compounds after their application on plants. In this proof‐of‐concept study, a single treatment with RPMs was able to maintain reduced disease severity even 20 days after application, results that were comparable to those observed for treatment with copper. Moreover, in a preliminary experiment related to the bees' survival assay, the RPMs were incubated in sucrose solution for up to three months and they were kept stable under these conditions. However, further studies should be performed to precisely explore the stability of RPMs *in planta*, in order to determine the optimal frequency of treatment application.

## Experimental procedures

### Synthesis of random peptide mixtures

Synthesis of the random peptide mixtures (RPMs) was carried out according to Hayouka *et al*. (2013). Briefly, RPMs were synthesized using microwave irradiation on Rink Amide resin (Substitution 0.53 mmol g^−1^, 25 μmol) in Alltech filter tubes. Coupling reactions were conducted with binary combinations of protected amino acids, with a freshly prepared stock solution that contained the protected amino acids in 1:1 molar ratio of l‐Phenylalanine with either l‐Lysine or d‐Lysine (50 μmol) of each amino acid, which were used for each coupling step. Upon completion of the synthesis (20 cycles for 20 mer peptide chain length), the RPMs were cleaved from the resin, resuspended in double‐distilled water (DDW), frozen in dry ice and lyophilized. RPMs were analysed by MALDI‐TOF to evaluate molecular weight and quality and by amino acid analysis.

### Bacterial strains and growth conditions

Bacterial strains used in this study are described in Table 1. Unless stated otherwise, all strains except *Pseudomonas syringae* pv. *tomato* (Pst) DC3000 were grown on nutrient agar (NA, Difco) or nutrient broth (NB, Difco). *Pst* DC3000 cells were grown on Kings B agar plates (King *et al*., 1954) and NB. All strains were grown in solid medium at 28°C for 48 h and were stored at −80°C in 25% glycerol.

### Assessment of minimal inhibitory concentration (MIC) and minimal bactericidal concentration (MBC) values

To determine the antimicrobial activity of FK‐20 and FdK‐20 RPMs, MIC and MBC values were measured for the strains described in Table 1. The MIC was determined using sterile 96‐well plates (Corning 3650) by a broth microdilution method as described by Hayouka *et al*. (2013). Briefly, bacteria were grown for 48 h in NB at 28°C with shaking (180 rpm). Then, the bacterial cultures were diluted in NB to an optical density at 600 nm (OD_600_) of 0.1 using a ThermoSpectronic (Genesys 10uv) spectrophotometer. One hundred microlitre aliquots were added to 100 μl of NB medium containing RPMs at various concentrations in each well. The plates were then incubated at 28°C for 24 h. Bacterial growth was determined by measuring the OD at 595 nm using a Tecan Infinite Pro Plate reader. The MIC values were the lowest concentrations of the peptide mixtures that caused inhibition of bacterial growth (Hayouka *et al*., 2013). MIC values were determined as the average obtained from three independent experiments, with three replicates per strain/concentration combination in each experiment. For determination of MBC values, at the end of MIC assays, 5 μl was taken from each well and plated on NA plates in triplicates. The MBC values were determined as the average lowest concentration of RPMs that rendered no bacterial growth (Mah, 2014). The highest concentration tested was 200 μg ml^−1^.

### Assessment of antimicrobial activity of the RPMs in the kohlrabi–*Xanthomonas campestris* pv. *campestris* (Xcc) pathosystem (black rot disease)

Kohlrabi (*Brassica oleracea*) cv. Konmar (Eden Zerahim) seeds were germinated in plastic pots (7 cm diameter, 9 cm height; one seed per pot) filled with a peat‐based commercial soil mixture (Shacham Givat Ada) and grown in a glasshouse at 26–28°C. Plants were used for inoculation after developing four fully expanded leaves (~4 weeks after sowing). Inoculation was carried out using a modification of the leaf‐clipping method (Valverde *et al*., 2007). Briefly, the three youngest fully expanded leaves of the plants were cut at two different sites (one at each side of the leaf, cuttings of about 1 cm length) with a scissor that was previously dipped in a solution containing 200 μg ml^−1^ RPMs (FK‐20 or FdK‐20) or 0.25% Kocide 2000 (DuPont), a broadly used agricultural bactericide that contains 53.8% (w/w) copper hydroxide. As a control, leaves were cut with a scissor that was previously dipped in sterile DDW. After 15 min, a suspension of *Xcc* ATCC 33913 at 10^6^ colony‐forming units CFU ml^−1^ (or sterilized DDW as control) was applied to the cuttings using a sterile swab stick. Bacterial concentrations in the inocula were verified by dilution plating. Treated plants were covered with a plastic bag for 48 h to increase relative humidity (thus promoting bacterial infection) and maintained in the glasshouse. Disease severity was assessed at several days postinoculation (dpi) according to the following scale: 0, healthy (no visible lesion); 1, slight yellowing up to 2 mm from the infection site; 2, yellowing ranging from 2 to 5 mm from the inoculation site; 3, lesion larger than 5 mm from the inoculation site, composed mainly by yellowing but showing appearance of brown necrosis; and 4, large brown necrosis around the inoculation site (Fig. 2A). In addition, we assessed whether RPM or Kocide treatment induced phytotoxic effects in leaves by following appearance of symptoms in plants that were not inoculated with bacteria. Three experiments were carried out. In each experiment, each treatment was applied on four plants (with a total of 12 leaves and 24 treated sites). Data were statistically analysed by ANCOVA (dpi as covariant) using JMP Pro 12 (SAS Institute).

### Assessment of antimicrobial activity of the RPMs in the tomato–*Xanthomonas perforans* pathosystem (bacterial spot disease)

Tomato (*Solanum lycopersicum*) cv. FA‐144 (also known as Daniela; Hazera Genetics) seeds were germinated in plastic trays containing vermiculite in a glasshouse at 26–28°C. Two weeks after sowing, the seedlings were transferred to plastic pots (10 cm diameter, 12 cm height; four seedlings per pot) filled with a peat‐based commercial soil mixture (Shacham Givat Ada). Plants were used for inoculation after they developed four fully expanded leaves (~4 weeks after transplant). The five most external leaflets of the three youngest fully expanded leaves were pretreated on both sides with solutions containing 200 μg ml^−1^ RPMs (FK‐20 or FdK‐20), 0.25% Kocide 2000 (DuPont) or sterilized DDW (control). The treatments were carried out by spraying an average of 0.17 ml of the solution per leaflet. Twenty‐four hours later, each leaflet was sprayed in both sides with ~0.18 ml of a suspension containing ~5 × 10^8^ CFU ml^−1^ of *X. perforans* 97‐2 (OD_600_ = 0.2). Bacterial concentrations in the inocula were verified by dilution plating. Inoculated plants were covered with a plastic bag for 24 h to increase relative humidity and maintained in the glasshouse. Disease severity in each leaflet was determined every 2–3 days after appearance of disease symptoms (~5 to 6 dpi) using the fallowing scale: 0, healthy leaflet; 1, less than 30 dark spots; 2, over 30 spots without yellowing or browning; 3, over 30 spots with slight yellowing or browning; 4, over 30 spots with significant browning/necrosis that covers less than 50% of the leaflet; 5, over 30 spots with significant browning/necrosis that covers more than 50% of the leaflet; and 6, more than 90% of the leaflet is necrotic (Fig. 3A). As similar as in kohlrabi experiments, we assessed whether RPMs or Kocide causes phytotoxic effects on treated leaves. Three experiments were carried out. In each experiment, each treatment was applied on six plants, and data were statistically analysed by ANCOVA (dpi as covariant) using JMP Pro 12 (SAS Institute).

### Assessment of effects of RPMs on honeybees

No‐choice dietary feeding studies were conducted to measure the effects of FK‐20 and FdK‐20 on survival and food consumption of honeybees (Robyn and Galen, 2007). At day 0, newly emerged bees were placed in Perspex cages (11 × 6 × 20 cm) with four feeding tubes, all filled with a treatment solution (Paoli *et al*., 2014) and four different feeders filled with bee‐collected pollen pellets mixture (Beit‐Itzhak). The bees were fed with a diet containing 40% sucrose syrup (Sigma) to which one of the following were added: FK‐20 (100 or 200 μg ml^−1^), FdK‐20 (100 or 200 μg ml^−1^) or control (no added peptides). Three replicate cages were assigned to each diet treatment, with 60–90 randomly selected bees per treatment from six source colonies. The bees were provided access to the sugar syrup and pollen only. Each cage was equipped with a sliding transparent front, which allowed daily counting and removal of dead bees and measurement of syrup consumption. Sugar syrup and pollen were filled as needed and replaced with fresh pollen and syrup once a week. Cages were kept in an incubator at 31 ± 1°C, 51 ± 10% relative humidity and 24 h at dark. Every 1–3 days, sugar syrup consumption was recorded by measuring the change in liquid level in each feeding tube between two sequential recordings (in mm). An adjustment for evaporation was made based on the data from feeding tubes in an empty cage. Daily sugar syrup consumption per bee was calculated by dividing the adjusted food consumption for each treatment by the number of bees still alive at the day of sampling. Pollen consumption was measured by dividing the weight loss of the pollen feeder by the number of live bees at the given, accumulated time. A pilot experiment with lower peptide concentrations was conducted prior to this experiment (data not shown). Data were statistically analysed by survival analysis and by one‐way analysis of variance (ANOVA)/Welch test using JMP Pro 12.

### Cytotoxicity towards mammalian cells

Caco‐2 (ATCC, HTB‐37) cells were grown at 37°C in a humidified atmosphere with 5% CO_2_ in Dulbecco's modified Eagle's medium (DMEM; Sigma) with 2 mM l‐glutamine, 1% (v/v) Penstrep and 20% (v/v) fetal bovine serum (Biological Industries). The cytotoxic potential of FK‐20 and FdK‐20 was determined following incubation of exponentially growing cells using the MTT assay. This method is based on the reduction in the tetrazolium salt, 3‐(4,5‐dimethylthiazol‐2‐yl)‐2,5‐diphenyltetrazolium bromide (MTT), into a crystalline blue formazan product by the cellular oxidoreductases of viable cells. The resultant formazan crystal formation is proportional to the number of viable cells. The cells were incubated with peptides at serial dilutions in the supplemented DMEM without phenol. Following 24 h incubation, cells were washed to remove test compounds and were then incubated with 50 μl of MTT solution in culture media (0.5 mg/ml) at 37°C in a humid atmosphere with 5% CO_2_ for 1.5 h. Fifty microlitres of MTT solution was used as blank. The media was then gently aspirated from test cultures, and 100 μl of dimethylsulphoxide (DMSO) was added to all wells. The plates were then incubated for 10 min at 37°C in dark conditions and the absorbance was read at 595 nm in a plate reader (Tecan Infinite Pro). The percentage of cell viability was calculated in comparison with control samples that were not exposed to RPMs. Percentage of cell viability values were determined as the average obtained from three independent experiments, with two replicates per RPM concentration in each experiment.

## Conclusions

Plant pathogenic bacteria are among the most important causal agents of plant diseases. The chemical control options to cope with this type of bacterial diseases are limited and often inadequate. Natural and rationally designed antimicrobial peptides (AMPs) might have the potential to serve as novel crop protection agents. In this study, we demonstrated that random peptide mixtures (RPMs), as unique AMP derivatives consisting of random combinations of l‐phenylalanine and l/d‐lysine, have strong antimicrobial effects *in vitro* and *in planta*. These RPMs were shown to possess the potential to be used as crop protection agents, also because their effect on reduction in disease severity was comparable to that exerted by a copper‐based, commercial bactericide, although at much lower concentration than the latter. Under tested conditions, the RPMs had no influence on the survival of bees and did not affect the bees' pollen and sugar syrup consumption. Further studies are needed to elucidate the mode of action of the RPMs and how they interact with the plant tissue and the environment, as well as to explore the potential application of this technology in other pathosystems. In addition, further experiments should be carried out to assess the stability of the compounds following their application in the field.

## Author contribution

ST synthesized the RPMs and carried out all experiments alone, or together with DTA and HBT (*in planta* experiments) and SS (effects of RPMs on honeybees). TS carried out the cytotoxicity of mammalian cells experiment. ST, ZH and SB conceived all experiments and wrote the manuscript.

## Conflict of interests

None declared.

## References

[mbt213258-bib-0001] Alan, A.R. , and Earle, E.D. (2002) Sensitivity of bacterial and fungal plant pathogens to the lytic peptides, MSI‐99, magainin II, and cecropin B. Mol Plant Microbe Interact 15: 701–708. 12118886 10.1094/MPMI.2002.15.7.701

[mbt213258-bib-0002] Badosa, E. , Ferre, R. , Planas, M. , Feliu, L. , Besalú, E. , Cabrefiga, J. , *et al* (2007) A library of linear undecapeptides with bactericidal activity against phytopathogenic bacteria. Peptides 28: 2276–2285. 17980935 10.1016/j.peptides.2007.09.010

[mbt213258-bib-0003] Behlau, F. , Canteros, B.I. , Minsavage, G.V. , Jones, J.B. , and Graham, J.H. (2011) Molecular characterization of copper resistance genes from *Xanthomonas citri* subsp. *citri* and *Xanthomonas alfalfae* subsp. *citrumelonis* . Appl Environ Microbiol 77: 4089–4096. 21515725 10.1128/AEM.03043-10 PMC3131652

[mbt213258-bib-0004] Burdman, S. , Kots, N. , Kritzman, G. , and Kopelowitz, J. (2005) Molecular, physiological, and host‐range characterization of *Acidovorax avenae* subsp. *citrulli* isolates from watermelon and melon in Israel. Plant Dis 89: 1339–1347. 10.1094/PD-89-1339 30791314

[mbt213258-bib-0005] Cuppels, D.A. (1986) Generation and characterization of Tn5 insertion mutations in *Pseudomonas syringae* pv. *tomato* . Appl Environ Microbiol 51: 323–327. 16346988 10.1128/aem.51.2.323-327.1986 PMC238867

[mbt213258-bib-0006] Datta, A. , Ghosh, A. , Airoldi, C. , Sperandeo, P. , Mroue, K.H. , Jiménez‐Barbero, J. , *et al* (2015) Antimicrobial peptides: insights into membrane permeabilization, lipopolysaccharide fragmentation and application in plant disease control. Sci Rep 5: 11951. 26144972 10.1038/srep11951 PMC4491704

[mbt213258-bib-0007] De La Fuente, L. , and Burdman, S. (2011) Pathogenic and beneficial plant‐associated bacteria In Encycl. Life Support Syst. Lal R. (ed). Oxford: EOLSS Publishers.

[mbt213258-bib-0008] Diamond, G. , Beckloff, N. , Weinberg, A. , and Kisich, K.O. (2009) The roles of antimicrobial peptides in innate host defense. Curr Pharm Des 15: 2377–2392. 19601838 10.2174/138161209788682325 PMC2750833

[mbt213258-bib-0009] Dobson, A.J. , Purves, J. , and Rolff, J. (2014) Increased survival of experimentally evolved antimicrobial peptide‐resistant *Staphylococcus aureus* in an animal host. Evol Appl 7: 905–912. 25469169 10.1111/eva.12184 PMC4211720

[mbt213258-bib-0010] Genersch, E. (2010) Honey bee pathology: current threats to honey bees and beekeeping. Appl Microbiol Biotechnol 87: 87–97. 20401479 10.1007/s00253-010-2573-8

[mbt213258-bib-0011] Gitaitis, R. , and Walcott, R. (2007) The epidemiology and management of seedborne bacterial diseases. Annu Rev Phytopathol 45: 371–397. 17474875 10.1146/annurev.phyto.45.062806.094321

[mbt213258-bib-0012] Güell, I. , Cabrefiga, J. , Badosa, E. , Ferre, R. , Talleda, M. , Bardají, E. , *et al* (2011) Improvement of the efficacy of linear undecapeptides against plant‐pathogenic bacteria by incorporation of D‐amino acids. Appl Environ Microbiol 77: 2667–2675. 21335383 10.1128/AEM.02759-10 PMC3126360

[mbt213258-bib-0013] Hancock, R.E. (2001) Cationic peptides: effectors in innate immunity and novel antimicrobials. Lancet Infect Dis 1: 156–164. 11871492 10.1016/S1473-3099(01)00092-5

[mbt213258-bib-0014] Hayouka, Z. , Chakraborty, S. , Liu, R. , Boersma, M.D. , Weisblum, B. , and Gellman, S.H. (2013) Interplay among subunit identity, subunit proportion, chain length, and stereochemistry in the activity profile of sequence‐random peptide mixtures. J Am Chem Soc 135: 11748–11751. 23909610 10.1021/ja406231b PMC3856984

[mbt213258-bib-3001] Hayouka, Z. , Bella, A. , Stern, T. , Jiang, H. , Ray, S. , Grovenor, C. , and Ryadnov, M. (2017) Binary encoding of peptide sequences into differential antimicrobial mechanisms. Angew Chemie Int Ed Engl 56: 8099–8103. 10.1002/anie.201702313 28557193

[mbt213258-bib-0015] Henry, M. , Béguin, M. , Requier, F. , Rollin, O. , Odoux, J.‐F. , Aupinel, P. , *et al* (2012) A common pesticide decreases foraging success and survival in honey bees. Science 336: 348–350. 22461498 10.1126/science.1215039

[mbt213258-bib-0016] Jones, J.B. , Woltz, S.S. , Jones, J.P. , and Portier, K.L. (1991) Population dynamics of *Xanthomonas campestris* pathovar *vesicatoria* on tomato leaflets treated with copper bactericides. Phytopathology 81: 714–719.

[mbt213258-bib-0017] Jones, J.B. , Lacy, G.H. , Bouzar, H. , Stall, R.E. , and Schaad, N.W. (2004) Reclassification of xanthomonads associated with bacterial spot disease of tomato and pepper. Syst Appl Microbiol 27: 755–762. 15612634 10.1078/0723202042369884

[mbt213258-bib-0018] King, E.O. , Ward, M.K. , and Raney, D.E. (1954) Two simple media for the demonstration of pyocyanin and fluorescin. J Lab Clin Med 44: 301–307. 13184240

[mbt213258-bib-0019] Lugo, A.J. , Elibox, W. , Jones, J.B. , and Ramsubhag, A. (2013) Copper resistance in *Xanthomonas campestris* pv. *campestris* affecting crucifers in Trinidad. Eur J Plant Pathol 136: 61–70.

[mbt213258-bib-0020] Mah, T.F. (2014) Establishing the minimal bactericidal concentration of an antimicrobial agent for planktonic cells (MBC‐P) and biofilm cells (MBC‐B). J Vis Exp 83: e50854. 10.3791/50854 PMC4047662 24430536

[mbt213258-bib-0021] Mahlein, A.K. , Oerke, E.C. , Steiner, U. , and Dehne, H.W. (2012) Recent advances in sensing plant diseases for precision crop protection. Eur J Plant Pathol 133: 197–209.

[mbt213258-bib-0022] Marcos, J.F. , Muñoz, A. , Pérez‐Payá, E. , Misra, S. , and López‐García, B. (2008) Identification and rational design of novel antimicrobial peptides for plant protection. Annu Rev Phytopathol 46: 273–301. 18439131 10.1146/annurev.phyto.121307.094843

[mbt213258-bib-0023] Meletzus, D. , and Eichenlaub, R. (1991) Transformation of the phytopathogenic bacterium *Clavibacter michiganense* subsp. *michiganense* by electroporation and development of a cloning vector. J Bacteriol 173: 184–190. 1898919 10.1128/jb.173.1.184-190.1991 PMC207173

[mbt213258-bib-0024] Mukherjee, S. , and Hooper, L.V. (2015) Antimicrobial defense of the intestine. Immunity 42: 28–39. 25607457 10.1016/j.immuni.2014.12.028

[mbt213258-bib-0025] Oren, Z. , and Shai, Y. (1997) Selective lysis of bacteria but not mammalian cells by diastereomers of melittin: structure‐function study. Biochemistry 36: 1826–1835. 9048567 10.1021/bi962507l

[mbt213258-bib-0026] Oren, Z. , Ramesh, J. , Avrahami, D. , Suryaprakash, N. , Shai, Y. , and Jelinek, R. (2002) Structures and mode of membrane interaction of a short α helical lytic peptide and its diastereomer determined by NMR, FTIR, and fluorescence spectroscopy. Eur J Biochem 269: 3869–3880. 12180963 10.1046/j.1432-1033.2002.03080.x

[mbt213258-bib-0027] Paoli, P.P. , Wakeling, L.A. , Wright, G.A. , and Ford, D. (2014) The dietary proportion of essential amino acids and Sir2 influence lifespan in the honeybee. Age (Dordr) 36: 9649. 24715247 10.1007/s11357-014-9649-9 PMC4082578

[mbt213258-bib-0028] Potnis, N. , Timilsina, S. , Strayer, A. , Shantharaj, D. , Barak, J.D. , Paret, M.L. , *et al* (2015) Bacterial spot of tomato and pepper: diverse Xanthomonas species with a wide variety of virulence factors posing a worldwide challenge. Mol Plant Pathol 16: 907–920. 25649754 10.1111/mpp.12244 PMC6638463

[mbt213258-bib-0029] Rathinakumar, R. , Walkenhorst, W.F. , and Wimley, W.C. (2009) Broad‐spectrum antimicrobial peptides by rational combinatorial design and high‐throughput screening: the importance of interfacial activity. J Am Chem Soc 131: 7609–7617. 19445503 10.1021/ja8093247 PMC2935846

[mbt213258-bib-0030] Robyn, R. , and Galen, P.D. (2007) Effects of Bt corn pollen on honey bees: emphasis on protocol development. Apidologie 38: 368–377.

[mbt213258-bib-0032] da Silva, A.C.R. , Ferro, J.A. , Reinach, F.C. , Farah, C.S. , Furlan, L.R. , Quaggio, R.B. , *et al* (2002) Comparison of the genomes of two *Xanthomonas* pathogens with differing host specificities. Nature 417: 459–463. 12024217 10.1038/417459a

[mbt213258-bib-0033] Stern, T. , Zelinger, E. , and Hayouka, Z. (2016) Random peptide mixtures inhibit and eradicate methicillin‐resistant Staphylococcus aureus biofilms. Chem Commun 52: 7102–7105. 10.1039/c6cc01438k 27161246

[mbt213258-bib-0034] Valverde, A. , Hubert, T. , Stolov, A. , Dagar, A. , Kopelowitz, J. , and Burdman, S. (2007) Assessment of genetic diversity of *Xanthomonas campestris* pv. *campestris* isolates from Israel by various DNA fingerprinting techniques. Plant Pathol 56: 17–25.

[mbt213258-bib-0035] Vicente, J.G. , and Holub, E.B. (2013) *Xanthomonas campestris* pv. *campestris* (cause of black rot of crucifers) in the genomic era is still a worldwide threat to brassica crops. Mol Plant Pathol 14: 2–18. 23051837 10.1111/j.1364-3703.2012.00833.x PMC6638727

[mbt213258-bib-0036] Walcott, R.R. , Langston, D.B. , Sanders, F.H. , and Gitaitis, R.D. (2000) Investigating intraspecific variation of *Acidovorax avenae* subsp. *citrulli* using DNA fingerprinting and whole cell fatty acid analysis. Phytopathology 90: 191–196. 18944608 10.1094/PHYTO.2000.90.2.191

[mbt213258-bib-0037] Worthington, R.J. , Rogers, S.A. , Huigens, W. , and Melander, C. (2012) Foliar‐applied small molecule that suppresses biofilm formation and enhances control of copper‐resistant *Xanthomonas euvesicatoria* on pepper. Plant Dis 96: 1638–1644. 10.1094/PDIS-02-12-0190-RE 30727459

[mbt213258-bib-0038] Zasloff, M. (2002) Antimicrobial peptides of multicellular organisms. Nature 415: 389–395. 11807545 10.1038/415389a

